# Insights into the Diagnostic Potential of Extracellular Vesicles and Their miRNA Signature from Liquid Biopsy as Early Biomarkers of Diabetic Micro/Macrovascular Complications

**DOI:** 10.3390/ijms18091974

**Published:** 2017-09-14

**Authors:** Valeria La Marca, Alessandra Fierabracci

**Affiliations:** Type 1 Diabetes Centre, Infectivology and Clinical Trials Research Department, Children’s Hospital Bambino Gesù, Viale San Paolo 15, 00146 Rome, Italy; valeria.lamarca@opbg.net

**Keywords:** extracellular vesicles (EVs), endothelial-derived microparticles, platelet-derived microparticles, non-invasive biomarkers, miRNAs signature, diabetes associated complications, micro- and macrovascular damage, diabetic nephropathy

## Abstract

Extracellular vesicles (EVs) represent a heterogeneous population of small vesicles, consisting of a phospholipidic bilayer surrounding a soluble interior cargo. Almost all cell types release EVs, thus they are naturally present in all body fluids. Among the several potential applications, EVs could be used as drug delivery vehicles in disease treatment, in immune therapy because of their immunomodulatory properties and in regenerative medicine. In addition to general markers, EVs are characterized by the presence of specific biomarkers (proteins and miRNAs) that allow the identification of their cell or tissue origin. For these features, they represent a potential powerful diagnostic tool to monitor state and progression of specific diseases. A large body of studies supports the idea that endothelial derived (EMPs) together with platelet-derived microparticles (PMPs) are deeply involved in the pathogenesis of diseases characterized by micro- and macrovascular damages, including diabetes. Existing literature suggests that the detection of circulating EMPs and PMPs and their specific miRNA profile may represent a very useful non-invasive signature to achieve information on the onset of peculiar disease manifestations. In this review, we discuss the possible utility of EVs in the early diagnosis of diabetes-associated microvascular complications, specifically related to kidney.

## 1. Introduction

In the complex scenario of a cell’s life cycle, scanned by differentiation, expansion, carrying out functions and programmed cell death, an incredible amount of stimuli continuously leads to the release of EVs. Physiological conditions, such as shear stress [1], cellular activation [2] or apoptosis [3] normally induce microvesiculation. In pathological conditions, as well as oncogenic transformation [4,5,6], inflammation [7,8,9] or other strong cellular stresses, this process is dramatically enhanced, with an increase in EVs production [10]. Vesicles release represents a highly conserved process in prokaryotes and eukaryotes suggesting the extent of a dynamic extracellular communication network, deeply involved in organ and tissue regulation [11]. This mechanism of cell-to-cell communication represents a necessary condition for proper coordination, both during development and among different cell types within adult tissues [12]. EVs are structures consisting of fluid surrounded by a phospholipidic bilayer, originated by mother cell membranes and contain a large variety of lipids and proteins. Membrane glycoproteins, distinctive of the parental cells, allow a fine identification of their origin (vide infra). In addition, EVs contain a soluble interior cargo composed by proteins and genetic material (mRNAs and micro RNAs (miRNAs)) [13]. During EVs generation, specific proteins may be included or excluded from the cell membrane, thus surface protein expression can be not identical to their parental cells. EVs were initially precipitated from platelet-free plasma (reviewed in [12]) although for many years they were considered inert cellular debris.

EVs are nowadays recognized as a heterogeneous population of circulating small vesicles originating from almost all cell types: endothelial cells (EC), monocytes, lymphocytes, platelets, leukocytes and erythrocytes, but also neurons, cancer and stem cells [14]. Furthermore, they are naturally present in body fluids including blood, saliva, urine, seminal fluid, nasal secretions, tears, synovial fluid, vitreous humor, cerebrospinal fluid and breast milk [14].

EVs have being largely studied for their therapeutic potential, since a system based on exosomes and microvesicles may represent a very potent tool for drug delivery, which even improves drug solubility and allows the passage across physiological barriers such as blood–brain barrier (BBB) [11,15,16]. Interestingly, in vitro experiments recently demonstrated EVs efficacy in reducing glioblastoma cell proliferation and induction of tumor cell apoptosis [17]. Moreover EVs, loaded with anti-blastic drugs, exerted an enhanced anti-tumor activity in vitro [17].

Besides their usage as drug delivery vehicles, EVs already revealed their potential application in immune therapy [18] because of their immuno-activatory or immuno-inhibitory properties. It is well known that they are responsible for the immunomodulatory effects of their mesenchymal stem cell (MSC) progenitors [19,20] by acting through paracrine mechanisms. Infected macrophages are able to secrete EVs containing pro-inflammatory pathogen-derived molecules [21]. Furthermore, mycoplasma-infected cultured cells release pro-inflammatory exosomes stimulating B- and T-lymphocytes [22]. EVs are also able to directly present antigens and contain MHC-peptide complexes for the initiation of immune responses by antigen-presenting cells (APCs) [23]. Exosomes produced by tumor-derived membranes may exert both an immuno-stimulatory effect, by transferring tumor antigens to dendritic cells (DCs) [4] and an immuno-inhibitory response, inducing T cell apoptosis in vitro [24,25,26]. Furthermore, safety and efficacy of the novel anti-tumor vaccine, i.e., α-type 1 polarized DCs (αDC1) loaded with synthetic peptides specific for glioma-associated antigen (GAA) epitopes, were demonstrated in a phase I/II vaccination trial [27]. Finally, several interesting pieces of evidence [28,29,30] ascribed the regenerative properties of MSCs to their EVs paracrine signals. These paracrine factors can influence both stem cell niches and tissue response on adjacent parenchymal and stromal cells, by enhancing cell survival, self-renewal and activating endogenous mechanisms for repair and regeneration (reviewed in [12]). As regards EVs disadvantages, their massive release by cancer cells contribute to extracellular matrix degradation, thus to invasive growth and angiogenesis, contributing to metastasis and horizontal propagation of oncogenes. Furthermore, EVs can lead cancer cells to escape from immune surveillance (vide supra) by exposing Fas ligand, the ligand for the death receptor Fas, promoting T cell apoptosis and inhibiting T cell adaptive immune responses (reviewed in [12]). In recent years, the scientific community has been focusing into the diagnostic and prognostic potential of EVs since they can provide a non-invasive and continuous signature to predict disease onset and monitor its progression [31]. In this review, we therefore discuss the possible utility of EVs and their associated miRNAs for the early diagnosis of diabetes mellitus (DM)-associated microvascular complications, with focus on renal damages.

## 2. An Overview on Microvesicles Biology

**Figure 1:**
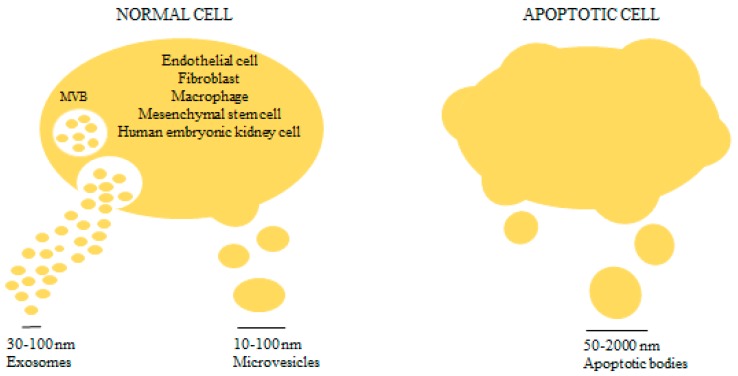
EVs mechanism of intercellular communication. Depending on the physical and chemical properties of the cell compartments of biogenesis, EVs show different dimensions.

EVs classification is based on their different sizes and biogenesis. “Microparticles” (MPs), also known as microvesicles or ectosomes, originate from the outward budding of the plasma membrane, “exosomes” are formed by fusion between endocytic vesicles and the plasma membrane and “apoptotic bodies” are generated by apoptotic cells [13] (Figure 1). These latter can be more abundant than exosomes or MPs under specific conditions and can vary in content among different biofluids [32]. The size of EVs depends, at least in part, by their origin; a lipid bilayer has a thickness of 5 nm, so that the smallest MP size is around 30 nm and the largest is around 1 μm. Endosomes, whose size ranges 200–500 nm, allow release of exosomes having a 30–100 nm diameter (Figure 1).

Among the general markers, most exosomes express proteins such as tetraspannins (CD9, CD63 and CD81), Alix, flotillin, TSG101 and Rab5b [33,34]. The enrichment in cholesterol, ceramide, sphingolipids and raft-associated phosphoglycerides, provides an additional tracking opportunity for exosomes characterization [35]. Cell activation and apoptosis, accompanied by an increase in cytosolic calcium, alter the normal distribution of phospholipids in the plasma membrane, due to inhibition of *flippase* activity, with a consequent increased phosphatidyl serine (PS) exposure on the outer leaflet of the membrane. PS externalization allows MVs identification, while specific protein markers additionally define the cell origin [36]. Blood cells and erythrocyte-derived MPs are identified by the presence of CD235a on their membrane [37,38]; CD4 and CD8 label lymphocytes-derived MPs [39,40,41,42]; PMPs are revealed by CD41 and CD42 [41,43,44]; and CD144 and CD146 are specific for EC [45].

EVs shedding is highly influenced by intracellular elements such as calcium, that affects membrane phospholipid distribution through specific enzymes, i.e., flippase, floppase and scramblase. Calcium ions also intervene in cytoskeleton reorganization (reviewed in [12]).

**Figure 2:**
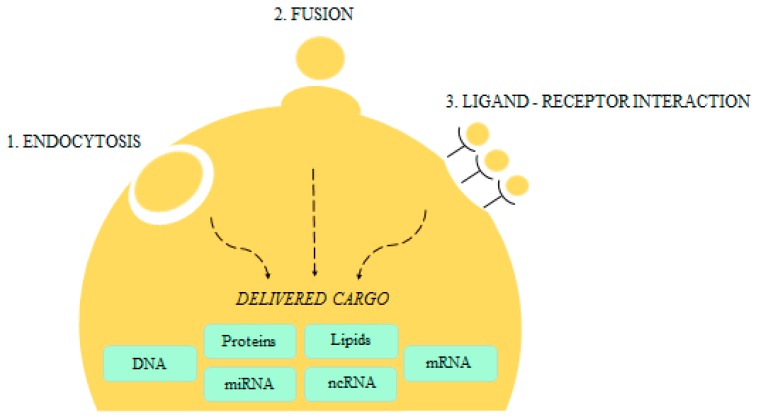
EVs mechanisms of intercellular communication without direct cell-to-cell contact.

Interactions between microvesicles and recipient cells can occur throughout different mechanisms (Figure 2) such as ligand-receptor binding, direct fusion with plasma membranes or uptake by recipient cells [19]. MVs uptake can occur via endocytic pathways such as phagocytosis, micropinocytosis, lipid-raft mediated internalization, clathrin-dependent or independent endocytosis [19]. Interaction between specific ligands on microvesicles surface and receptors on target cells leads to the activation of intracellular signaling pathways. Nevertheless, many EVs, once released from a cellular element, may rapidly break down, thus releasing extracellularly their content. EVs represent a novel mechanism through which cells exchange genetic information since nucleic acids are protected within their membranes from plasma ribonucleases (reviewed in [12]). Remarkably, EVs are able to induce epigenetic changes of neighboring cells by horizontal transfer of RNA.

## 3. EVs Diagnostic Potential

The study of EVs is opening new horizons for their potential application not only as therapeutic tools but also as clinical biomarkers for monitoring disease progression (vide supra) [10,13]. Even if most clinical data derive from studies of tumor patients, increased levels of EVs have been detected in body fluids in a variety of cardiovascular and inflammatory pathologies, obesity, atherosclerosis, diabetes and metabolic syndrome (vide infra), as well as in infectious and neurodegenerative diseases including Alzheimer’s, Parkinson’s diseases and multiple sclerosis [46,47,48,49,50]. Furthermore, in recent years, special attention was focused on miRNAs, a group of small, single-stranded, non-coding RNAs, deeply involved in the regulation of gene expression by post-transcriptional interference with complementary mRNAs [51]. EV-associated specific miRNA profiles were found putatively correlated with peculiar pathological conditions when assayed in biological fluids such as plasma, sera and urine [52,53]. Indeed, circulatory cell-free miRNAs are easily detectable and very stable due to the protection from RNase degradation, being embedded in exosomes, microvesicles or apoptotic bodies [54] or through formation of protein–miR complexes with Argonaute 2 (Ago2) or high-density lipoprotein (HDL)-associated proteins [55,56].

### EVs Quantification Issues

EVs isolation from cell culture supernatants and from body fluids [57] has been essentially performed by differential steps of centrifugation, aimed to recover sequentially pelleted smaller particles [57,58]. Nevertheless, to date, EVs quantification from liquid biopsies represents an open challenge that requires a reliable standardization. Due to their small size, the conventional methods used for cell quantification cannot be applied to EVs. The most utilized methods for the analysis of EV quantity, size and features are represented by transmission electron microscopy (TEM), flow cytometry (FACS), nanoparticle-tracking analysis (NTA), and Tunable Resistive Pulse Sensing technology (TRPS). Total protein content, varying among different EVs subtypes, cannot be considered an accurate method because of a possible contamination by high molecular weight proteins [32].

Every single measurement method is based on different physical principles leading, therefore, to the determination of different radius values [13]. Electron microscopy uses electrons to generate an image with a resolution down to the nanometer, and allows evaluating structure and morphology of cell-secreted vesicles [13]. TEM technique requires fixation, dehydration and staining of biological samples before imaging; these treatments may dramatically damage the vesicles and affect their size and morphology. Flow cytometry is a valid method to study EVs both in physiological and in pathological conditions, but its sensitivity is often insufficient to visualize smallest EVs [59]. NTA is another technique that measures size distribution of EVs within a 50–1000 nm range. This tool allows the direct visualization of scattering particles irradiated by a laser beam: the hydrodynamic radius of every single particle is calculated by the analysis of its Brownian motion [13,60], with the advantage of a lower detection limit compared to flow cytometry, both in plasma and in supernatants of cultured cells [61,62]. TRPS principle relies on ionic flow disruption at the time particles pass through a single nanopore separating two fluidic cell compartments [32].

The establishment of a set of EVs markers, indicative of their cell or tissue origin, could be useful for the quantification of specific vesicle subsets in biological samples and their potential disease correlation [12]. This issue has been especially unraveled for EMPs and PMPs.

## 4. Relevance of EMPs and PMPs

Endothelium is a thin layer of flat epithelial cells that limits serous cavities, lymph and blood vessels, and acts as a selective barrier in the continuous exchange of molecules between blood and tissues [63,64]. The endothelium of some tissues and organs, such as kidney or liver, is characterized by discontinuities or fenestrations between cells, large enough to allow the passage of large molecules or proteins. In other organs, EC are joined together by different types of adhesive cell-to-cell junctions, formed by transmembrane molecules linked to cytoskeletal or cytoplasmic proteins that selectively allow passage of water, macromolecules and even blood cells [65]. Vascular EC are largely involved in the regulation of normal vascular tone and permeability, homeostasis maintenance, coagulation/fibrinolysis balance, composition of subendothelial matrix, leukocytic diapedesis and thrombogenesis prevention [66]. EC are able to exert their multiple functions by releasing several regulatory mediators (nitric oxide, prostanoids, endothelin, angiotensin II, tissue-type plasminogen activator (t-PA), and plasminogen activator inhibitor-1 (PAI-1)), adhesion molecules and cytokines [45]. A pathological event such as dyslipidemia, hyperglycemia or inflammation occurring in several conditions (vide supra) may modify natural endothelial properties inducing cell activation thus endothelial dysfunction [66]. Dysfunctional EC release vasoactive substances, EMPs and chemotactic factors altogether contribute to the initiation of inflammatory response and to eventual atherogenic development [67,68,69,70]. Besides activated EC, apoptotic EC may also release EMPs having a different surface immunophenotype [71,72,73]. In detail, activated cell-derived MPs express a high amount of CD62E, while apoptotic EMPs are mainly CD31+ [74,75]. An elevated ratio of CD31+/Annexin V+ EMPs to CD62E+ EMPs reflects an impaired immune phenotype of EMPs and allows to diagnose through a specific pattern of EMPs the origin and degree of endothelial dysfunction in dysmetabolic disorders [66,76].

High plasma levels of EMPs have been found in patients with hypertension [77], hypertriglyceridemia, acute coronary artery disease (CAD) [78], peripheral vascular disease [79] and DM [80]. Endothelial dysfunction cannot be considered a clear hallmark of the diabetic state, rather a key factor in the pathogenesis of athero-thrombogenic complications, retinopathy, nephropathy, atherosclerosis [9,81], and micro- and macroangiopathy. In several studies, CD144 (VE cadherin) positive MPs have been identified as specific EC particles, the increase of circulating CD144 EMPs represents a very specific marker of EC dysfunction, and could be useful to identify DM patients with risk of CAD [9]. In a recent study, Fan et al. (2016) [82] pointed out that EMPs are involved in the activation of platelet vesiculation.

PMPs together with EMPs have also been widely investigated for their involvement in inflammation; coagulation; diseases characterized by the impairment of vascular function, such as atherosclerosis, diabetes, and hypertension; and in connective tissue diseases [83,84]. Nomura et al. (2004) [85] observed that PMPs are able to promote interaction between EC and monocytes in patients with Type 2 diabetes (T2D), therefore they were potentially implicated in the onset of diabetes-associated complications. As assessed by Tsimerman et al. (2011) [86] EVs from diabetic patients, especially from those with diabetic foot, show a high pro-coagulant activity. PMPs were even found significantly elevated in pediatric Type 1 diabetes (T1D) patients, particularly in association with early microvascular complications [83] (vide infra).

## 5. EVs and EVs-Associated mRNAs Diagnostic Potential in Diabetes and Its Complications

DM is the most relevant metabolic disorder, affecting about 100 million persons worldwide, with a strong trend to increase. Classically, DM is classified in Type 1 (T1D) and Type 2 (T2D). T1D, also known as insulin-dependent DM, and representing 5–10% of cases, is an autoimmune multifactorial disorder occurring in human leukocyte antigen (HLA) genetically-predisposed individuals as a consequence of organ-specific immune destruction of the insulin-producing β cells in the islets of Langerhans within the pancreas [87,88,89]. It is generally recognized that T1D derives from a breakdown in immune regulation that leads to expansion of autoreactive CD4+ and CD8+ T cells, autoantibody-producing B lymphocytes and activation of the innate immune system [88]. T2D, or non insulin-dependent DM, characterized by insulin resistance, accounts for 90–95% of cases. It is a complex metabolic disorder, of heterogeneous etiology with contributing social, behavioral and environmental risk factors [90]. The number of affected patients is expected to double during the next 20 years [91].

DM leads to chronic complications, such as accelerated development of cardiovascular diseases, end-stage renal disease, loss of visual acuity and limb amputations, the main cause of morbidity and mortality in DM affected individuals [90]. Many data support the idea of a close connection between duration and severity of diabetes and micro/macrovascular damage [74] including coronary, cerebrovascular and peripheral arterial disease (PAD) due to complex dysfunction of main components of the vascular compartment [92,93,94,95].

Initial studies unraveled the utility of PMPs and EMPs as diagnostic markers in diabetes. Although EVs quantification/characterization remains an open challenge within the scientific community (vide supra), increased plasmatic levels of PMPs and CD62P/CD63 positive platelets were found in patients with DM compared to normal controls. These novel markers correlated with hypercoagulability, suggesting the utility of antiplatelet therapy, i.e., cilostazol, to prevent the development of complications, especially nephropathy, in patients with poor blood glucose control [96].

Based on the use of specific markers for characterization, Tsimerman et al. (2011) [86] demonstrated that PMPs and EMPs and negatively charged phospholipid-bearing MPs were at highest levels in T2D patients with severe foot ulcers. The same result was obtained by Lakthter et al. (2015) [97] in T1D patients, who exhibited higher levels of PMPs and EMPs, total Annexin V-positive blood cell MP (TMP) and TMP procoagulant activity. Furthermore, the last parameter correlated with HbA1c and dysglicemia. Instead, in T2D patients there was only an increase of TMP without the increase in procoagulant activity [45].

In the study by Sun et al. (2017) [98], levels of urinary CD63-positive exosomes were found increased at early stage of renal injury in 62 early diabetic nephropathic (DN) subjects [98]. Nevertheless, CD63 expression was significantly increased in normoalbuminuric patients rather than in the microalbuminuric group, probably due to a weak compensatory increase in GFR at an early stage.

Several investigators addressed the issue of identifying a specific dysregulated plasma miRNA signature in either T2D and obese patients or T1D affected subjects in order to depict novel biomarkers of diagnostic utility. Table 1 focused on most frequently detected miRNAs and EV-associated miRNAs emerging from an extensive literature review (Table 1).

MiR-126, highly enriched in EC and in platelets, is one of the miRNAs more frequently investigated for its relevance in endothelial homeostasis, in maintaining vascular integrity, in angiogenesis and in wound repair. When released by EC, miR-126 modulates VEGF (vascular-endothelial growth factor) responsiveness, thus contributing to vascular protection in a paracrine manner. As endothelial activation and inflammation are hallmarks of micro- and macrovascular complications in diabetes, loss of miR-126 was considered predictor as well as risk estimation/classification marker not only for early diabetes but also for endothelial dysfunctions due to diabetes [99]. As VEGF is a crucial mediator in DN, miR-126 could be helpful also in predicting this type of complication (vide infra). Furthermore, miR-126 could represent a candidate marker for monitoring the efficacy of miRNA-based therapeutic intervention of vascular complications related to the disease [100]. Coming to the analysis of relevant manuscripts, Zampetaki et al. (2010) [100] provided a detailed plasmatic mi-RNA signature in a large population-based cohort, the Bruneck study. This was initially designed to investigate the epidemiology and pathogenesis of atherosclerosis and later extended to all major human diseases, including T2D. Reduced levels of miR-126 were observed, and correlated to peripheral artery disease in T2D. The same aberrant miRNA expression was observed by Barutta et al. (2016) [99] in an extensive analysis of more than 400 serum samples of T2D patients and healthy subjects. In other studies, Olivieri (2015) [101] and Jansen (2016) [102] confirmed a reduction of miR-126 in T2D patients. In particular, Jansen et al. [102] found that loss of miR-126 is related to CAD risk.

Unlike T2D, the role of miR-126 in T1D is not yet fully clarified. Osipova et al. (2014) [103] for the first time analyzed blood and urine samples of T1D pediatric patients, focusing on miRNAs known to have relevance in diabetes and cardiovascular/renal damages. Regarding miR-126, no differences emerged in plasmatic T1D samples, while lower miR-126 levels were confirmed in urine T1D samples compared to controls (vide infra).

The same authors focused their studies also on miR-21, a profibrotic miRNA in cardiovascular diseases [109], known to induce fibrosis in many organs including heart and kidney [109,110] and involved in endothelial-to-mesenchymal transition [111]. MiR-21 was upregulated both in plasma and urine samples of pediatric T1D patients [103]. MiR-21 upregulation was also proposed as useful biomarker for already existent fibrotic remodeling. Furthermore, the positive correlation emerged in urine samples between miR-21 and the inflammatory C-reactive protein (CRP) suggesting the presence of ongoing inflammatory events in the kidney of T1D patients [103]. Regarding T2D, Olivieri et al. (2015) [101] confirmed higher plasma levels of miR-21 in diabetic patients with cardiovascular complications.

MiR-29 is another relevant miRNA involved in diabetes and its complications. MiR-29 family is composed of miR-29a, miR-29b and miR-29c, sharing the same seed sequence. The most important function of miR-29 consists in its protective role in fibrotic disease, including kidney fibrosis [112]. MiR-29 is also involved in the pathogenesis of DN in diabetic mice [113,114]. Furthermore, miR-29 is upregulated in muscle, fat and liver in type 2 diabetic rats and caused insulin resistance in adipocytes [115]. An increase in miR-29 levels is found in the serum of T1D children [105] and adult patients with T2DM [105]. Both hyperglycemia and pro-inflammatory cytokines, the hallmarks of DM, upregulated the expression of miR-29 family miRNAs [113,116], and the suppression of miR-29 with anti miR-29 oligomers protected against DN [113]. Additional studies highlighted the presence of a wide spectrum of putative miRNAs useful as DM biomarkers. The Bruneck study [100] revealed lower plasma levels of miR-20b, miR-15a, miR-191, miR-197, mi-223, miR-320 and miR-486, while a modest upregulation of miR-28-3p even at an early stage [100]. A downregulation of miR-191, parallel to miR-200b, was shown by Dangwal et al. (2015) [117]. Plasmatic miR-150, -192, -27a and -320a were found specifically upregulated by Karolina et al. (2012) [106] both in metabolic syndrome and T2D. MiR-320a, together with miR-27b, was found upregulated and associated to diabetic retinopathy also by Zampetaki et al. (2016) [107]. Furthermore, the authors detected miR-17, -197, -509-5p, -92a and -320a in plasmatic exosomes, with a similar expression pattern as in whole blood, supporting the hypothesis that circulating cell-free miRNAs are packaged into exosomes [107]. Pescador et al. (2013) [118] demonstrated that miR-138 or miR-376a could be a useful predictive tool for distinguishing obese patients from healthy controls, diabetics and obese diabetics. In particular the combination of miR-503 and miR-138 could discriminate diabetics from obese diabetics. A decreased serum level of miR-146a was indicated as a potential marker of chronic inflammation in T2D patients by Baldeon et al. (2014) [119]. Santovito et al. (2014) [120] discovered a significant upregulation of miR-326, -186, -532-5p, -127-3p, and a significant downregulation of let-7a and let-7f in plasma of T2D patients compared to controls. Other miRNAs candidates as diabetes and diabetic complications biomarkers are represented by miR-103 [121], miR-18a and miR-34c [122], miR-222, miR-let7d, miR-139 miR-199 and miR-26a [102], miR-24 [100,108], miR-454-3p, miR-222-3p, miR-144-5p and miR-345-3p [123].

## 6. Potential Role of EVs and their MiRNAs Profiles in the Prediction of Diabetic Renal Complications

DN represents one of the most relevant chronic complications of DM [124] and the major cause of end-stage renal failure [125]. The number of patients with chronic renal damage due to DN is dramatically increased over the past decades [96] mostly due to the incidence of obesity and T2D in developed countries [126]. Metabolic and hemodynamic alterations as well as inflammation underlie DN development. Early blood pressure changes within the kidney and impairment of glomerular microcirculation, leading to glomerular hypertrophy and sclerosis, are critical in DN progression [126]. At present, clinical biomarkers including glomerular filtration rate (GFR), proteinuria and urinary sediment evaluation can help to identify etiology of chronic kidney disease but do not allow a specific diagnosis neither clarify disease staging [127]. Therefore, the finding of non-invasive biomarkers could obviate the use of kidney biopsy, a procedure implying complication risks, and could improve diagnostic accuracy. To this extent, the best source of biomarkers to unravel renal damage in diabetes is represented by urine. An easy and non-invasive analysis of miRNAs contained in urinary exosomes has recently been proposed in several studies in order to monitor early renal complications, since their dysregulated levels have been detected in urine of human diabetic patients (reviewed in [127]). Table 2 focuses on most frequently detected miRNAs and EV-associated miRNAs in diabetic patients affected by renal complications following an extensive literature review (Table 2).

In initial investigations, Szeto et al. (2012) [128] found lower miR-15 levels in association with diabetic glomerulosclerosis, and an increased level of miR-17 in patients with IgA nephropathy. Furthermore, lower levels of miR-21 and miR-216a in urinary sediments correlated with a faster decline of renal functions [128]. Conversely increased levels of miR-21 and miR-210 in plasma and urine samples of T1D pediatric patients were reported by Osipova et al. (2014) [103]. In the last report, urinary miR-126 levels were significantly lower in diabetic patients than in age- and gender-matched controls (vide supra). This miRNA concentration negatively correlated with HbA1c levels, suggesting a damaging effect driven by long-term high plasma glucose. It was demonstrated that miR-126 is expressed in glomerular and peritubular EC targeting SPRED1 (Sprouty-related, EVH1 domain containing protein) and PIK3R2 (phosphoinositol-3 kinase regulatory subunit 2), i.e., negative repressors of VEGF pathway [132] (vide supra). These phenomena envisage that decreased levels of miR-126 are associated with reduced response to VEGF and endothelial dysfunction.

As detailed in Table 2, several other miRNAs were highlighted in other investigations carried out with the aim to precisely characterize and quantify EVs and EV-associated miRNA profile predictive of diabetic complications. As stated before, an easily available biological sample such as urine represents a major advantage. A close association between single miRNA variation and renal diabetic complications was always depicted in spite of variability on reported miRNAs specificities. A unique urinary minimum signature related to diabetes complications remains to be fully validated.

Reduced urinary levels of miR-192 were found in nephropathic patients characterized by diabetic glomerulosclerosis, and increased levels of miR-200c were detected in patients with minimal change nephropathy and with focal glomerulosclerosis [129]. By the analysis of Barutta et al. (2013) [130], miR-130a and miR-145 were found enriched in diabetic patients with microalbuminuria while miR-155 and miR-424 were decreased compared to normoalbuminurics and non-diabetic controls.

Peng et al. (2013) [131] focused their studies on miR-29 family (consisting of miR-29a, miR-29b, miR-29c, vide supra) involved in DN pathogenesis, and proposed miR-29 as biomarker for DN and atherosclerosis in T2D patients. By analyzing 83 T2D patients, urinary miR-29a and miR-29c were significantly higher compared to miR-29b. Furthermore, urinary miR-29a was significantly increased in patients with albuminuria [131] than in normoalbuminurics. miR-29b correlated with carotid intima-media thickness in T2D patients. Other putative DN biomarkers were identified in other studies. As regards miR-619, -486-3p, -335-5p, -552, -1912, -1224-3p, -424-5p and -141-3p [133], miR-320c and miR-6068 [134] were found upregulated, while miR-2861, miR-1915-3p and miR-4532 were downregulated in DN patients [127]. Increased levels of serum miR-217 were correlated with the development of proteinuria in T2DN patients [135]. Urinary exosomal miR-133b, miR-342, and miR-30a [136] and miR-192 [137] were expressed at significantly higher levels in T2DN patients compared to normal.

## 7. Conclusions and Future Perspectives

In recent years, the scientific community has been debating methods for EVs isolation, characterization and quantification. The expensive and complex procedures being used so far need to be further improved to feasibly distinguish different EV subpopulations in highly pure EV preparations. A more defined standardization of these technological tools could lead to easier downstream characterization of EVs by transcriptomic, miRnomic and proteomic platforms to accurately define “selective diagnostic panels of markers” for disease prediction, staging and progression. Nevertheless, it needs to be pointed out that the translational significance of results that could be obtained by novel technologies always strictly relies on the appropriate selection of biological material from a homogeneous cohort of patients with same clinical characteristics, stage of disease and ethnic origin.

In light of the foregoing extensive discussion of the existing literature, we can easily envisage that EVs and their miRNA cargo from liquid biopsies represent, nowadays, non-invasive biomarkers with great potential in longitudinal investigations related to several disease conditions including DM. In particular, EVs detection in urine could especially improve prediction by introducing non-invasive renal signatures of early onset and progression of microvascular renal damage in DM without the need for invasive diagnostic or radiological procedures. Nevertheless, future studies will clarify the precise cause-effect link between dysregulation of EV-related miRNAs and DN, and the precise role of these small non-coding RNA in the progression of diabetic complications [126].

## Figures and Tables

**Table 1:** MiRNAs and EV-associated miRNAs in T1D and T2D. Most frequently detected dysregulated miRNAs in T1D and T2D patients with disease-associated complications.

miRNA	Level	Confirmed EVs Association	Complications	Ref.	Type of Diabetes
126	↓		VEGF resistance, endothelial dysfunction, inflammation	Zampetaki et al., 2010 [100]	T2D
				Barutta et al., 2016 [99]	T2D
				Osipova et al., 2014 [103]	T1D
				Jansen et al., 2016 [102]	T2D
				Olivieri et al., 2015 [101]	T2D
21	↑		Kidney inflammation	Osipova et al., 2014 [103]	T1D
			Cardiovascular damages	Olivieri et al., 2015 [101]	T2D
29 (29a, 29b, 29c)	↑			Nielsen et al., 2012 [104]	T1D
				Kong et al., 2011 [105]	T2D
27a	↑			Karolina et al., 2012 [106]	T2D
27b, 320	↑	Present	Retinopathy	Karolina et al., 2012 [106], Zampetaki et al., 2016 [107]	T2D
24	↓			Zampetaki et al., 2010 [100]	T2D
				Deng et al., 2017 [108]	T2D

**Table 2:** miRNAs and EV-associated miRNAs in patients with diabetic renal involvement. Most frequently detected miRNAs signature detectable in urine of patients with DN at different stages of disease.

miRNA	Level	Confirmed EVs Association	Renal Complications	Ref.
15	↓		Diabetic glomerulosclerosis	Szeto et al., 2012 [128]
17	↑		IgA nephropathy	
21, 216a	↓		Renal functions decline	
638	↓		Diabetic nephropathy	Wang et al., 2013 [129]
192	↓			
200c	↑		Diabetic nephropathy, glomerulosclerosis	
			Minimal change nephropathy, focal glomerulosclerosis	
130a, 145	↑	Present	Microalbuminuria	Barutta et al., 2013 [130]
155, 424	↓	Present		
29a	↑		Diabetic nephropathy, albuminuria	
29c	↑		Diabetic nephropathy	Peng et al., 2013 [131]
126	↓		Preclinical kidney disease, renal fibrosis	Osipova et al., 2014 [103]
21, 210	↑			

## Abbreviations

EVs	extracellular vesicles
MPs	microparticles
EMPs	endothelial-derived microparticles
PMPs	platelet-derived microparticles
EC	endothelial cells
DM	diabetes mellitus
T1D	type 1 diabetes
T2D	type 2 diabetes
miRNA	microRNA
DN	diabetic nephropathy
CAD	coronary arterial disease
CHD	coronary heart disease
PAD	peripheral arterial disease
VEGF	vascular endothelial grow factor
GFR	glomerular filtration rate
MVs	microvesicles
PS	phosphatidyl serine
CRP	C-reactive protein
